# Measuring Fatigue and Fatigability in Spinal Muscular Atrophy (SMA): Challenges and Opportunities

**DOI:** 10.3390/jcm12103458

**Published:** 2023-05-14

**Authors:** Rafael S. Rodriguez-Torres, David Uher, Emma L. Gay, Giorgia Coratti, Sally Dunaway Young, Annemarie Rohwer, Robert Muni Lofra, Darryl C. De Vivo, Michio Hirano, Nancy W. Glynn, Jacqueline Montes

**Affiliations:** 1 Department of Neurology, Columbia University Irving Medical Center, New York, NY 10032, USA; 2 Department of Rehabilitation and Regenerative Medicine, Columbia University Irving Medical Center, New York, NY 10032, USAjm598@cumc.columbia.edu (J.M.); 3 Department of Biobehavioral Sciences, Teachers College, Columbia University, New York, NY 10115, USA; 4 Department of Epidemiology, School of Public Health, University of Pittsburgh, Pittsburgh, PA 15213, USAepidnwg@pitt.edu (N.W.G.); 5 Pediatric Neurology Unit, Catholic University, 00135 Rome, Italy; 6 Centro Clinico Nemo, U.O.C. Neuropsichiatria Infantile Fondazione Policlinico Universitario Agostino Gemelli IRCCS, 00168 Rome, Italy; 7 Department of Neurology and Clinical Neurosciences, Stanford University School of Medicine, Palo Alto, CA 94305, USA; 8 Dubowitz Neuromuscular Centre, UCL Great Ormond Street Institute of Child Health, London WC1N 1EH, UK; 9 The John Walton Muscular Dystrophy Research Centre, Translational and Clinical Research Institute, Newcastle University and Newcastle Hospitals NHS Foundation Trust, Newcastle upon Tyne NE1 7RU, UK

**Keywords:** fatigue, fatigability, spinal muscular atrophy, patient-reported outcome measure, perceived fatigability

## Abstract

Fatigue, a common symptom, together with the characteristic of performance fatigability, are well-documented features of SMA that impact quality of life and function. Importantly, establishing associations between multidimensional self-reported fatigue scales and patient performance has proven difficult. This review was conducted to evaluate the various patient-reported fatigue scales applied in SMA, with the objective of considering the limitations and advantages of each measure. Variable use of fatigue-related nomenclature, including conflicting terminology interpretation, has affected assessment of physical fatigue attributes, specifically perceived fatigability. This review encourages the development of original patient-reported scales to enable perceived fatigability assessment, providing a potential complementary method of evaluating treatment response.

## 1. Introduction

### 1.1. SMA Background

Spinal muscular atrophy (SMA) is a rare autosomal recessive neuromuscular disorder that is caused by a deletion in the survival motor neuron (*SMNI*) gene, leading to SMN protein deficiency. It primarily results in the degeneration of alpha motor neurons in the spinal cord and brainstem. The *SMN2* gene serves as a backup, producing low levels of full-length SMN protein. Though imperfect, the number of *SMN2* copies has historically been associated with the severity of disease [1]. SMA is classified into types, defined by age of disease onset and highest developmental milestone achieved [2]. Clinical features of SMA include progressive skeletal muscle weakness and loss of motor and bulbar function [3,4].

The natural history of SMA and standards of care recommendations have been impacted by significant developments in available therapeutics [4,5]. There are currently three disease-modifying treatment options, whose availability varies based on several factors that are explained in detail elsewhere [6,7]. Nusinersen (Spinraza, Biogen Inc., Cambridge, MA, USA) is an antisense oligonucleotide therapy delivered intrathecally to target the *SMN2* gene and increase full-length SMN protein production [8]. Risdiplam (Evrysdi, F. Hoffmann-La Roche, Basel, Switzerland), a small molecule delivered orally, is also a splicing modulator that targets *SMN2* [9,10]. Onasemnogene abeparvovec-xioi (Zolgensma, Novartis Gene Therapies, Inc., Bannockburn, IL, USA) is an AAV9-based gene therapy delivered as a one-time intravenous administration to deliver a copy of *SMN1* [11]. A range in clinical response to available treatment options exists, with age at treatment initiation being a strong predictor of treatment efficacy [12].

### 1.2. Fatigue in SMA

Similar to other neurological and neuromuscular disorders, fatigue is widely known to be one of the most disabling symptoms of SMA [13,14,15]. The previously reported prevalence of patient-reported fatigue in SMA has ranged from 81 to 100% [14]. Despite advances demonstrated with SMN targeted therapies, fatigue remains a burdensome and challenging manifestation of SMA [13,16,17]. Importantly, the task of better studying and comprehending the real-life impact of interventions has been emphasized by several stakeholders [18,19,20,21]. Thus, a transition from strictly performance-based assessments towards new evaluation methods reflective of patient interests and experiences, such as a greater understanding of fatigue, is now in demand [18,20,21,22,23,24].

### 1.3. Conflicting Terminology

**Figure 1:**
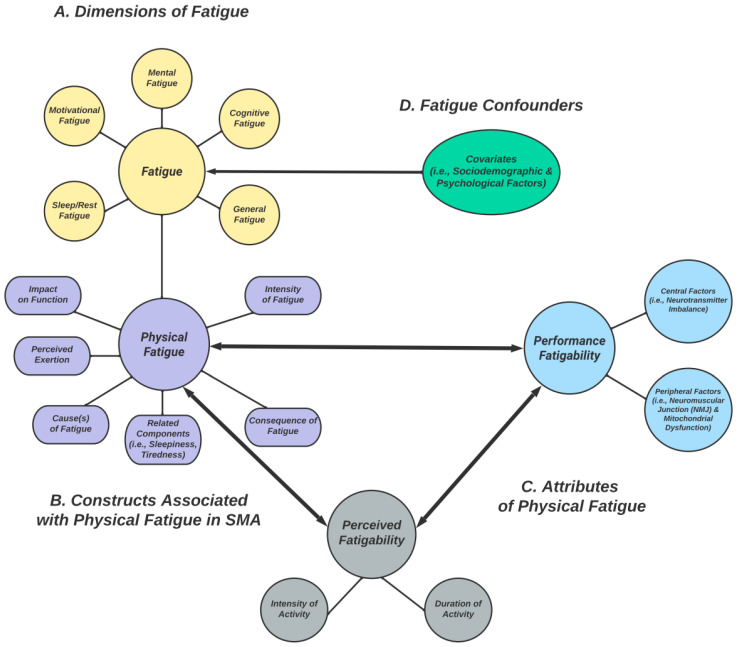
(**A**) Different dimensions of fatigue commonly studied in SMA (yellow). (**B**) Constructs related to the physical fatigue dimension, which are assessed by some PROs (purple). (**C**) Attributes of physical fatigue: (1) performance fatigability (light blue) and (2) perceived fatigability (gray). (**D**) Fatigue confounders (green). Two-way arrows represent relationships; they provide a theoretical framework for conceptualizing (physical) fatigue, performance fatigability, and perceived fatigability as interrelated concepts that likely interact and influence each other during physical activity.

It is important to reflect on the alternative fatigue-related terms frequently and, occasionally, incorrectly applied in the literature. For example, fatigue is an all-encompassing term that has been referred to as “global fatigue.” This catch-all phrase has, at times, been considered inclusive of patient-reported fatigue across fatigue dimensions. In SMA research, the most common fatigue dimensions studied are physical, general, mental/cognitive, sleep/rest, and motivational fatigue (Figure 1A). Fatigability, an attribute of physical fatigue is often referred to as “peripheral fatigue” or “physiological fatigue,” highlighting the physical dimensions pertinent to this measure. As perceived fatigability has not been as well defined in the SMA literature, it is often misconstrued to be synonymous with “perceived fatigue,” especially when those perceptions pertain to a physical task. However, this assumption is problematic for a multitude of reasons, chiefly related to the complexity of generalized fatigue self-report, even when it focuses on physical performance. The lack of consistent nomenclature makes it apparent that, at the very least, there is a collective acceptance and understanding of the multidimensional features of fatigue [25,26]. For the purpose of this review, multidimensional refers to the analysis of more than one element or dimension, of which either may be present concurrently [25,26]. 

### 1.4. Operational Definitions

The inconsistent terminology and assessment of fatigue dimensions and related constructs continues to impede progress in better understanding this unmet medical need in SMA. Since there is no accepted definition of fatigue and its subcomponents, it is important to understand the scope in which this paper was written, and which definitions were used. Fatigue is broadly described as a multidimensional subjective “overwhelming sense of tiredness, lack of energy, feeling of exhaustion, mental, physical or both” [27,28,29]; it can be measured at a particular point in time, capturing momentary perceptions (state-level fatigue), or in a manner where its chronic characteristics are highlighted (trait-level fatigue) [30]. Clinically, fatigue is most often quantified with questionnaires that require respondents to recall the perceived functional impact of experienced fatigue (perceived fatigue) on the ability to perform cognitive, physical, and/or psychosocial tasks. Complementary, fatigability is described as “work capacity”; it can be estimated (perceived fatigability) or quantified (objective fatigability). Objective fatigability is further characterized as the decrement in performance with a repeated task (performance fatigability) [27]. Perceived fatigability, understood as an attribute of the physical dimension of fatigue, refers to whole-body susceptibility to fatigue with physical activity of fixed intensities and durations [27,31,32,33]. Various measures of perceived fatigue and performance fatigability are validated in SMA. However, perceived fatigability has not been well studied.

## 2. Current Landscape and the Need for Alternative Outcome Measures

Though improvements in motor function and performance fatigability have been demonstrated with SMN-augmenting therapies, patient-reported improvements in fatigue have been shown to be fleeting [17,34,35]. Importantly, associations between patient-reported fatigue across dimensions and performance fatigability and motor function have been difficult to establish. As such, it is proposed that these concepts be considered distinct from each other, as factors unrelated to disease etiology tend to explain more of the variance observed on self-reported fatigue scales [13,27,30]. In a rapidly changing environment, where conventional SMA classifications are no longer appropriate and where patients are maintaining or improving function differently from their natural history, responsive patient-reported outcome measures (PROMs) are required [36]. As current fatigue assessment scales do not reflect functional status, the challenge of ameliorating relationships between patient-reported and performance fatigability and function is consequential. A recent scoping review by Slayter et al. (2022) found that few PROMs have been developed specifically for SMA. Thus, it is vital that novel assessments are created to improve understanding of the multifaceted burden of SMA.

## 3. Patient-Reported Fatigue

### 3.1. Dimensions of Fatigue Studied in SMA 

Fatigue is a complex concept that encompasses different dimensions and causalities, some of which are oftentimes secondary manifestations of disease processes and not directly related to the pathophysiology of disease [17,30]. There is an abundance of self-report questionnaires developed to assess health-related quality of life (HRQoL) areas, including fatigue, across neurological conditions, some of which have been used in SMA. These instruments exhibit several properties and benefits and measure various fatigue dimensions [22]. Additional constructs related to individual fatigue dimensions are captured by some scales, including severity of fatigue, consequences of fatigue symptomatology, and impact on function (Table 1). Among these many scales, only the Fatigue Severity Scale (FSS), and, more recently, the Multidimensional Fatigue Inventory (MFI), the Spinal Muscular Atrophy Health Index (SMA-HI), *PROfuture*, and SMA-TOOL scales have been validated in SMA, to some degree [22,37,38,39]. Other, non-SMA-specific scales have been used to assess perceived fatigue, including the PedsQL™ Multidimensional Fatigue Scale (PedsQL *MFS*) and the Patient-Reported Outcomes Measurement Information System Fatigue Short Form (PROMIS F-SF) questionnaire [13,40,41,42]. The multidimensional basis of these scales is valuable but makes it challenging to assess patient impressions on their susceptibility to fatigability, specifically.

### 3.2. Physical Fatigue Dimension

Fatigue is often described as a symptom of disease reflecting a formed interpretation of the specific condition [29]. In a scoping review by Billones et al. (2021), they describe how this symptom is regularly understood in a physical capacity; the lack of physical energy becomes an anchor construct that acts as a starting point for describing the fatigue experience. As SMA is principally considered a condition affecting physical concepts (i.e., muscle weakness and atrophy, decreased exercise capacity), it is understandable that most fatigue PROMs have primarily focused on the physical dimension of fatigue and related constructs. Further, this physical fatigue dimension has been proposed to perhaps be the most responsive to available treatments [38]. Interestingly, some have reported that less affected individuals, such as those with more *SMN2* copies, report greater fatigue [16,38]. However, this should be scrutinized, as inquiries related to fatigue with currently used questionnaires are often focused on activities most pertinent to stronger patients or are generally too broad and based solely on functional abilities. Further, cut-off values for clinically meaningful fatigue levels have been used inconsistently within and across scales [17]. 

Thus, the multidimensionality and ambiguity of analyses related to fatigue perceptions require that assessment methodology be enhanced. Billones et al. (2021) further found that few papers distinguished fatigue components using subscales that focused on distinctions between mental, physical, cognitive, and motivational elements impacting fatigue reports. They concluded that this generalized approach could limit the reliability of assessing these independent dimensions of fatigue [25]. Thus, developing validated, disease-specific scales to measure physical fatigue and related attributes in SMA is vital, both clinically and in research [60]. The psychometric properties of such scales should reflect a common conceptualization of physical fatigue and performance fatigability, the factors that influence this characteristic in SMA, as well as a dependable way of assessing patient perceptions of fatigability with daily activity [25].

## 4. Current Fatigue Scales and What They Measure

### 4.1. Fatigue Severity Scale (FSS)

The FSS is a nine-item multidimensional scale covering the physical, social, and cognitive effects of fatigue experienced during the “past week.” In SMA, it has been proposed to be more unidimensional when items 1 and 2 are omitted [39,43]. Item 1 queries the consequences of being fatigued, and item 2 queries what *might* cause fatigue, while the remaining items concern the experience of being fatigued. Scores range from 1 = strongly disagree to 7 = strongly agree, with higher scores representing greater fatigue. Scores of >4 and >5 indicate “abnormal fatigue” and “severe fatigue,” respectively [43]. Werlauff et al. (2014) reported that perceived fatigue in patients with SMA type II was shown to be captured using the FSS, with test–retest consistency demonstrated. Further, they reported the correlation between FSS and VAS-F to be 0.71 (Spearman’s rho) in SMA II patients. Dunaway Young et al. (2019) found that more than half of patients with SMA type II/III reported having abnormal/severe fatigue when using this scale and found no correlations between FSS and performance fatigability or function in untreated SMA. Despite improvements in maximal oxygen consumption (VO2max), Montes et al. (2015) showed no significant changes in FSS scores during a 6-month strength and aerobic exercise intervention in adult ambulatory patients. Kizina et al. (2020) demonstrated a moderately negative correlation between FSS and 6MWT after 6 months of treatment with nusinersen (Spinraza, Biogen Inc., Cambridge, MA, USA) in ambulatory adults with SMA. However, FSS scores 10 months after treatment initiation showed no difference from baseline [17]. In a longitudinal study looking at the patient-perceived benefits of salbutamol, Giovannetti et al. (2016) reported improvements in FSS scores 1-year post-treatment initiation. However, no correlation to functional performance was found, and they reported “recall bias” and “motivation to present their experience in more positive light” as potential confounders [45]. 

### 4.2. PedsQL™ Multidimensional Fatigue Scale (PedsQL™ MFS) 

PedsQL™ is a modular instrument designed to measure HRQoL and disease-specific symptoms in individuals aged 2–49 [46,48]. The 18-item PedsQL™ *MFS* was designed to measure fatigue symptomatology via child and adolescent self-reports, as well as parent-proxy reports [61]. It comprises the General Fatigue Scale (six items), the Sleep/Rest Fatigue Scale (six items), and the Cognitive Fatigue Scale (six items). Inquiries relate to the severity of a problem each item has presented during the past month. A 5-point Likert response scale is utilized (0 = never a problem, 4 = almost always a problem), with reverse scoring and linear transformation implementation resulting in a 0–100 scale (0 = 100, 1 = 75, 2 = 50, 3 = 25, 4 = 0); with higher scores indicating lower fatigue symptoms. Dunaway Young et al. (2019) found that all patients with SMA type II/III reported having fatigue (70–73%) when using this scale. However, they found that the subdomains of the PedsQL™ *MFS* had no relationship to function, quality of life, or fatigability in SMA [13]. Similar to the lack of significant changes in FSS scores following an exercise intervention described above, PedsQL™ *MFS* scores did not demonstrate significant changes in pediatric patients despite modest improvements in exercise capacity [44]. 

### 4.3. Multidimensional Fatigue Inventory (MFI)

The MFI contains 20 items categorized into five dimensions: *general fatigue, physical fatigue, mental fatigue, reduced motivation, and reduced activity* [49]. It measures how a patient feels “lately” on a 5-point Likert scale. A total score is calculated for each dimension, which ranges from four to 20 (higher scores = increased fatigue). Binz et al. (2021) found that 75% of adults with SMA were “abnormally fatigued,” and they identified *physical fatigue*, followed by *general fatigue*, and *reduced activity*, as the most relevant dimensions. Further, they postulated that as patients with SMA “are not able to perform exercise in the classical sense,” the MFI may be a more sensitive fatigue measure in SMA [16]. Though correlations between quality of life and *physical* and *general fatigue* subscales were identified, no associations were found between MFI fatigue subscales and motor function or functional rating scales in their validation study [22]. Moreover, although the most prevalent fatigue dimensions tended to decrease, prevalence rates and other multidimensional fatigue measures using the FSS and MFI fluctuated during 14 months of nusinersen (Spinraza, Biogen Inc., Cambridge, MA, USA) treatment. As natural history data on fatigue among SMA patients does not exist, they recommended longer observation periods and proposed that future efforts should focus on the *physical fatigue* dimension as a PROM to complement motor function-based assessments [16]. 

### 4.4. Patient-Reported Outcomes Measurement Information System Fatigue Short Form (F-SF)

The PROMIS Fatigue universal item banks assess a range of self-reported symptoms over the previous seven days. The 7-item PROMIS Fatigue-SF questionnaire relates to various fatigue dimensions and fatigue’s influence on decreasing one’s ability to execute daily activities, including the impact of family and social roles. Questions are divided into the experience and impact of fatigue on mental, physical, and social activities. Response options are on a 5-point Likert scale, 1 = *never* to 5 = *always*. Total scores range from 7–35, with higher totals indicating greater fatigue. Pediatric (8–17 years), adult (ages 18+) and parent-proxy short forms are available [41]. The parent-proxy short form has been utilized to assess perceived fatigue in SMA [40]. Belter et al. (2020) concluded that results from the PROMIS F-SF, used as part of their quality-of-life survey, appeared to “under-represent” the burden of fatigue often reported by patients and their caregivers. This inference was informed by the fact that upon converting PROMIS F-SF scores to T-scores, the converted scores among patients were <1 SD (range = 56–59 across SMA types) above the general population mean (T-score = 50). Furthermore, they pondered whether this may have had to do with the lack of sensitivity in the questions associated with fatigue, including their irrelevance to the SMA population [40]. The need to develop “SMA-specific” outcomes, with activities of significance based on the functional abilities of patients with SMA, was thus concluded. 

## 5. Additional Assessments of Physical Fatigue Related Construct(s) in SMA (Figure 1B)

### 5.1. Rating of Perceived Exertion (RPE)

The OMNI rating of perceived exertion (RPE) and the fatigue visual analog scale (VAS) have been commonly used as proxies for performance fatigability, including in SMA [16,53,56]. The OMNI RPE is a measure of perceived effort related to task performance [51,52]. This scale has a category rating format containing both pictorial and verbal descriptors along with a numerical response range, 0–10. Each pictorial descriptor is consistent with its corresponding verbal descriptor, from 0 = “Extremely easy” to 10 = “Extremely hard.” In SMA, the use of RPE has been found to lack associations with performance fatigability as measured on the 6MWT [54]. Cheng et al. (2022) concluded that the RPE should thus not be used as a substitute for performance fatigability but rather as a measure of the patient’s experience during exercise. Though perceived effort is likely associated with perceived fatigability, further qualifiers are needed, including task dependency, for example, to understand exertional differences with varying tasks. Still, perceived effort alone does not provide a complete picture of the perceived mechanical work (perceived fatigability) required to complete a specific task and should not be used as such. 

### 5.2. Fatigue Visual Analog Scale (VAS-F)

The fatigue VAS is a 100 mm horizontal line evaluating the intensity of fatigue between the anchors “no fatigue” and “extreme fatigue,” which captures feelings of fatigue at a particular point in time (state-level fatigue). It is regularly used to assess concurrent validity among fatigue questionnaires assessing the impact of fatigue on function. [39] As Werlauff et al. (2014) noted, this assumption is questionable given the different questions being addressed, intensity of fatigue versus impact of fatigue on participation. Further, patient-reported intensity of fatigue can be influenced by a multitude of factors, including those irrelevant to the task at hand. Indeed, Enoka et al. (2021) described state-level fatigue as perceptions that are derived from the interplay of interoceptive feedback mechanisms regarding the homeostatic state of the body. They report that these signals are also involved in other perceptions, cognitions, actions, and emotional behaviors [30]. As a result, one cannot assume that at a particular point in time, asking vague questions about the intensity of fatigue with an activity can be truly indicative of the estimated work capacity that was necessary to complete that activity. The impact of factors (i.e., motivation, emotions, pain) unrelated to the task, but which nonetheless influence mechanical output, cannot be fully removed from the patient’s report using this type of scale. A study examining a novel goal attainment approach to fatigue reduction in multiple sclerosis (MS) found that both patients and healthy controls reported increased VAS (state-level) fatigue only in the “no reward group” [62]. Further, a significant decrease in state-level fatigue occurred in both groups only when presented with a rewarding (monetary) outcome [62]. These findings highlighted the motivational elements influencing this type of patient report. Thus, the VAS should not be used in isolation when evaluating fatigue in SMA. van der Heul et al. (2022), in a study assessing mastication endurance via the Six-Minute Mastication Test (6MMT) in ambulant and non-ambulant individuals with SMA, found that fatigue-VAS was significantly higher directly after performance as well as 5 min post-testing [56,63]. Importantly, no correlation analysis between performance fatigability (6MMT) and fatigue-VAS was performed. As it relates to potential state-level fatigue confounding variables, pain was also reported to be high after test completion [56].

### 5.3. SMA-Health Index (SMA-HI)

The SMA-HI is a multifaceted, disease-specific PROM to assess SMA patients’ perceptions of disease burden across 15 areas of health, including fatigue [57,59]. It includes 107 symptomatic questions, representing 14 themes, with an additional supplemental item bank for ambulatory individuals. Each subscale is represented on a 0–100 scale (0 representing no disease burden). The total SMA-HI score is a weighted sum of its subscale scores (0–100). Seven questions relate to the symptom of fatigue and its impact on function, which were found to have a prevalence of >90% across SMA types [23]. In a recent study using the SMA-HI, fatigue subscale scores were found to be worse in non-ambulatory versus ambulatory patients [58]. To our knowledge, correlation analyses between fatigue subscale scores on the SMA-HI and performance fatigability have not been performed. 

### 5.4. SMA-TOOL

The SMA-TOOL was designed to assess patient- and caregiver-oriented measurements in SMA. The fatigue section included questions borrowed from the Neuro-QoL Fatigue Computer Adaptive Test (CAT), which is a self-report scale to assess fatigue and its impact on function in neurological populations [64]. As part of the SMA-TOOL, an additional 10–11 items developed by the Fundación Atrofia Muscular España (FundAME) were included to assess perceptions of fatigability. Notably, none of the fatigue-related questions inquire about perceived discrepancies between effort expended and ability to complete the action in question. Results from their study in untreated SMA patients identified perceived fatigability as being the most impacted domain, though they also found that perceived fatigability scores did not change in the predicted way with respect to clinical global impression-improvement scores (CGI-I). Patients who had “much improved” or “minimally improved” reported higher perceived fatigability scores [37]. Furthermore, they reported that perceived fatigability scores strongly worsened with disease severity, suggesting a motor neuron reserve capacity relationship to perceived fatigability [37].

### 5.5. PROfuture

The *PROfuture* is a recently developed questionnaire designed to assess “physical fatigue and perceived fatigability” in SMA [38]. Questions refer to the presence of fatigue symptoms during the past seven days. Physical fatigue inquiries address five aspects: the need to rest during the day, the need to select activities, difficulty maintaining posture, loss of energy during the day, and the presence of prolonged tiredness after making a greater effort than usual. Questions related to perceived fatigability assess the respondent’s experience with “inability to finish a task once started,” meant to reflect efforts that require sustained and repeated use of the upper or lower limbs. Three possible answer options include “never or almost never/sometimes/always or nearly always.” In their pilot study, Domine et al. (2022) found that more than half of the patients surveyed reported symptoms of physical fatigue, though the frequency and type of symptoms varied between functional groups: non-sitters, sitters, and walkers. A higher frequency of perceived fatigability in the upper limbs in non-sitters (74–100%), followed by sitters (22–95%), then walkers (17%), was reportedly observed. We acknowledge that an assessment of the “inability to complete a task once started” gets closer to a true perceived fatigability evaluation. However, this perceived inability cannot be measured in any specific manner, as again, the individual sense of effort required to perform the activity cannot be quantified. Specifically, there is a lack of standardized intensity and duration anchors, which would allow for comparisons within and across individuals.

## 6. Attributes of Physical Fatigue (Figure 1C)

### 6.1. Performance Fatigability

Performance fatigability and the notion of alterations in motor output due to repetitive muscle contractions have been well documented in SMA. It is primarily measured by quantifying the decline in one or more aspects of performance during continuous activity or prolonged tasks. It is the opposite of “endurance,” which involves the maintenance of constant, self-regulated power or velocity [27]. In SMA, performance fatigability and diminished endurance have been demonstrated with the use of validated performance-based assessments, such as the Six-Minute Walk Test (6MWT) and Endurance Shuttle Tests (ESTs), including the Endurance Shuttle Walk Test (ESWT), as well as the Nine Hole Peg Test (ESNHPT) and Box and Block Test (ESBBT) for the upper extremities in non-ambulatory individuals [53,65,66,67]. For those with bulbar dysfunction, the 6MMT has been recently used to demonstrate performance fatigability in the muscles of mastication [56,63]. However, the mechanisms responsible for the increased levels of fatigability in SMA cannot be explained by muscle weakness alone [19,22,28]. Previous work has proposed that disabling fatigability in SMA may be caused by an activity-dependent conduction block (ADCB) caused by collateral sprouting [14]. Others have shown neuromuscular junction (NMJ) dysfunction, caused by maturation and developmental abnormalities due to SMN insufficiency [68,69,70]. More recently, the role of mitochondrial dysfunction and its impact on reduced exercise capacity have been demonstrated [71,72]. Nonetheless, it is recognized that performance fatigability is a characteristic of the SMA disease process [73,74]. 

### 6.2. Perceived Fatigability

While the self-report measures of fatigue illustrated above are important in highlighting various elements of fatigue and their prevalence in SMA, it is difficult to assess patients’ perceptions regulating specific physical activity performance using any of these scales. Though the SMA-TOOL and the *PROfuture* scales have intended to develop self-report batteries to assess perceived fatigability with repetitive tasks, the assumptions made about assessing this attribute are undetermined. Both scales describe perceived difficulty with completing tasks and further emphasize that tasks of increasing difficulty tend to be performed by stronger patients. Though the difficulty in task completion may be related to the trait fatigability in SMA, the extent of this susceptibility to fatigability is not captured as the questions are not anchored to measurable activity modifiers (i.e., intensity and duration). Moreover, the use of a parent proxy to report on patient experience, as was allowed in the SMA-TOOL validation study, is discouraged in SMA. Previous work has shown that discrepancies exist between parents and children regarding their perceived degree of illness [75]. As such, the assessment of perceived work capacity with functional tasks should be exclusively reflective of the patient’s experience. We disagree with the interpretation made by Binz et al. (2021) that lack of exercise performance in a “classical sense” in SMA means a more generalized approach should be taken when inquiring about the patient’s physical condition. An improved solution would be to develop scales with activities and topics relevant to the SMA population. 

## 7. Challenges and Opportunities

### 7.1. Confounders of Patient-Reported Fatigue 

Various psychological factors influence fatigue perceptions, across dimensions, in several disease states, and among healthy individuals. These include task familiarity, temperament, motivation, and others [27,76]. In Parkinson’s disease (PD), depressed mood has been shown to be a confounder of mental, physical, and generalized fatigue [77]. In SMA, sociodemographic and psychological factors appear to impact how an individual experiences fatigue (Figure 1D). Compared to unemployed individuals, employed individuals with SMA have been shown to report less fatigue on the MFI, FSS, and SMA-HI scales [22,59]. Further, participants with <12 years of education were found to experience more *general* fatigue on the MFI, as also seen in the general population. In the category of *mental fatigue* on the MFI, depression/anxiety were further found to be confounding [16]. Researchers have acknowledged that these findings may indeed be an effect that is independent of SMA disease etiology [17,22]. These personal factors, while important in understanding and addressing them, thwart the measurement of vulnerability to fatigability with specific activities. Fatigue-related phenomena, including sleepiness, tiredness, and apathy, which can be resulting symptoms of neurologic disease, can also skew reported measures of fatigue across dimensions, puzzling subjective treatment responses in SMA [16,78,79,80,81]. For example, patients with SMA frequently endure reduced pulmonary capacity, which leads to episodic sleep desaturation symptoms, and resultant daytime tiredness [17]. Though one can contextualize this as partly due to the sequalae of disease and understand its implication on patient energy, evaluation of daytime tiredness alone does not substitute for perceived fatigability assessment. 

### 7.2. Inadequate Reflection of Functional Status

There is a consistent absence of association between patient-reported fatigue assessed with available measures and patient performance. This is not only true in SMA but has been demonstrated in other conditions, such as MS, as well as conditions where fatigability is not so closely related to disease etiology, i.e., idiopathic inflammatory myopathies, myotonic dystrophies, and others [13,82,83,84]. The missing link has been suggested to be related to the underlying construct(s) that fatigue scales evaluate. Thus, modifying the way in which perceived fatigability scales are designed so that the construct being analyzed is commensurate with the objective measures of fatigability should be considered. Kluger et al. (2013) proposed that to improve perceived fatigue assessment, one should be intentional about the domain of performance being examined and the task used to induce the patient response being evaluated. They further explain that though contributory factors, such as arousal, may influence performance across multiple domains, perceptions of fatigue will be different among physical and cognitive tasks [27]. Thus, the sensitivity and specificity of a proposed perceived fatigability measure matter. Context related to the type of activities being used to assess perceived fatigability is important, along with inquiring about the estimated work capacity to execute the activity in question. A first step towards creating an appropriate perceived fatigability scale requires normalizing activities to be relevant across SMA functional levels. 

### 7.3. Central and Peripheral Factors and Relationship to Fatigability

There are several metabolic, hormonal, and signaling pathway stimuli that may induce the sensation of muscle fatigue, both centrally and peripherally. From a homeostatic standpoint, fatigue perceptions may be better understood within the context of energy regulation [27]. The accumulation of ammonia, increases in serotonin, and decrements in dopamine in the central nervous system (CNS) are potential contributors to homeostatic changes [30]. Evidence of biochemical cerebrospinal fluid (CSF) abnormalities in SMA, including reduced energy-related molecules, has recently emerged [85]. Though the role of these neurometabolic changes and their relationship to fatigue and fatigability have not been studied in SMA, these molecular changes in amino acids involved in the synthesis of neurotransmitters may be of relevance to central fatigability factors in SMA. 

Peripherally, at the neuromuscular junction (NMJ) and myofiber, anatomical and physiological disruptions of synaptic transmission, as well as energy dysregulation due to depletion of muscle glycogen, lactate accumulation, and ATP availability, may impact fatigue perceptions [30,74]. NMJ dysfunction has been demonstrated, where nerve conduction studies via repetitive nerve stimulation (NCS-RNS) showed abnormal decremental responses in about 49% of patients with types II/III SMA [68]. Additionally, Pera et al. (2017) identified NMJ dysfunction in 9/15 ambulatory patients who completed the 6MWT, revealing a strong association between changes in distance walked and RNS % decrement [69]. Deafferentation of motor neurons has been suggested to be an early event leading to primary motor neuron dysfunction [86]. Functionally, there is conflicting evidence regarding motor unit reserve capacity during physical tasks in SMA [65,87]. Nonetheless, the underpinnings of fatigability in SMA are likely multi-factorial [53]. 

Though traditionally understood as a motor neuron disease, the lack of SMN protein in non-neuronal cell types continues to emerge [88,89]. Muscle tissues from patients with SMA have demonstrated down-regulation of co-factors associated with mitochondrial biogenesis [71,90]. Diminished muscle oxygen uptake was also demonstrated in patients during maximal and submaximal exercise [72]. Similarly, metabolic impairments, including fatty acid metabolism and glucose intolerance, were shown in SMA mouse models [91]. Taken together, these are elements of the disease process that may influence the characteristic diminished endurance and fatigability of SMA. 

## 8. Proposal for Unidimensional Perceived Physical Fatigability Scale Development

Understanding how an individual processes and experiences these impairments in energy-regulating systems in the context of physical activity is fundamental. It is notable to highlight the distinctions in currently available SMN-directed interventions and their unknown, and perhaps selective, effects on the energy-implicated structures and tissues mentioned above (i.e., NMJ, muscle, and CSF). For example, Kim et al. (2020) demonstrated a motor neuron cell-independent result of selective SMN depletion in the muscle, which triggered SMA pathology in mouse models. These findings demonstrated that SMN protein may be intrinsically critical to muscle function and that repletion of SMN limited to the CNS only may ultimately lead to a chronic and insidious late-onset muscle condition [92]. Thus, the evaluation of subjective therapeutic treatment effects may provide further insight into responsiveness to various forms of SMN repletion therapies. The possibility exists that an individual’s perceptions of fatigability may be more sensitive to change than what has been demonstrated via the use of performance fatigability measures, which are limited only to patients who have the capacity to perform these assessments. Therefore, a patient-reported assessment may be increasingly valuable in measuring perceived fatigability’s real-life impact. 

Ultimately, research utilizing careful measurements of perceived energetic requirements to complete activity may be a better way of examining the impact of intervention on homeostatic-related factors in SMA. As general perceptions of fatigue may be overlayed with covariates unrelated to SMA pathology, it becomes difficult to interpret individual treatment responses as due to causative treatments in the form of SMN repletion. Importantly, the objective should not be to ignore the multifactorial aspects of physical fatigue report. However, by converging on perceived fatigability more precisely, we encourage a step towards better exploring this attribute of physical fatigue. With the advent of innovative therapeutics, complementary outcome measures other than motor function-based assessments are necessary. A PROM that anchors activities to intensity and duration can be useful in understanding an individual’s vulnerability to perceived fatigability, as has been demonstrated with the Pittsburgh Fatigability Scale [33]. 

### The Pittsburgh Fatigability Scale (PFS)

The PFS is a validated PROM designed to measure perceived fatigability in older adults. It has been found to demonstrate high concurrent and convergent validity against measures of objective (performance) fatigability, mobility, fitness, and physical function [33]. In their development of the PFS, Glynn et al. (2015) expanded on the concept of perceived fatigability by further categorizing it based on specific activities of a given intensity and duration. They explain that this conceptualization helps to present a more objective approach to measuring the degree to which fatigue limits someone physically. They identify deficiencies in using existing self-report tools as they are unable to capture the intensity and duration of stimuli required to initiate varying degrees of estimated performance abilities [33]. Perceived fatigability with activity of varying intensity and duration is a relevant concept when considering the proposed causative factors of fatigability in SMA. As these putative variables are largely related to systems implicated in energy production and regulation, perceived fatigability scales anchored to activity and duration may provide an improved method of assessing subjective responsiveness to treatment.

## 9. Discussion

The assessment of patient-reported fatigue has been repeatedly emphasized as a valuable outcome measure for research and clinical care [19]. However, consistent associations between disease severity influences such as age, SMA type, or ambulatory status and patient-reported fatigue have not been established [13,22,37]. This may be related to the idea that general perceptions of fatigue can have several contributing factors, including confounders unrelated to disease pathology, which currently applied PROMs fail to characterize [13].

It is evident that fatigue self-report is a multi-faceted, multi-factorial, and multidimensional symptom, as described by the different PROMs discussed in this review [25]. This multidimensionality is reflected in the method by which fatigue is queried; some scales focus on the cause of fatigue, and others focus on its impact on function across daily life, health, and general well-being. Importantly, each scale offers insight into the disruption that fatigue has in SMA, stressing that it is a symptom of disease that we should better aim to understand. It is also clear that fatigue reporting is sensitive to context and that meaning is influenced by individual experiences [25]. As such, it is difficult to compare fatigue reports across scales as the dimensions of fatigue included in each PROM vary. The physical fatigue dimension appears to be most relevant in SMA and is proposed to be most related to the pathophysiology of disease [38]. However, even the assessment of physical fatigue across the PROMs covered in this review is inconsistent. 

Perceived fatigability, defined as a whole-body measurement of a person’s susceptibility to fatigue related to physical performance, offers a more responsive measure of the degree to which fatigue limits physical function [33]. The sensitivity of such a scale is highlighted in the application of activity intensity and duration as key distinguishers. This method allows for changes in perceptions of fatigability to be captured in a more standardized way. Thus, a disease-specific PROM that prompts self-reflection regarding perceived fatigability in this manner may more closely resemble the evaluation of performance fatigability in SMA, which captures the decrement in motor output/performance over a specified period. 

## 10. Conclusions

The assumption that fatigue-related terminology is known to all has created confusion and a lack of consistency in assessment, both of which impede translation and result in delayed patient-centered treatment [27]. Most PROMs have been repurposed to fit the SMA population and broadly categorize function [36]. However, the expansive heterogeneity of SMA, including evolving phenotypes, necessitates that we continue to explore alternative methods to assess treatment response through the development of innovative PROMs. In this attempt towards developing validated and reliable scales, acknowledging concrete operational definitions of fatigue and its subdimensions is significant, as is creating disease-specific measures. Here, we propose a unidimensional approach to measuring an attribute of physical fatigue, namely perceived physical fatigability. The development of such a scale to better isolate and assess perceived physical fatigability in SMA is essential. This will expand insight into the continued unmet therapeutic needs regarding individual treatment response and its impact on daily function [19].

## Figures and Tables

**Table 1:** PROMs assessing different fatigue dimensions in SMA.

Clinical Measure (Scale Type)	Target Population	Author(s) in SMA	Dimension(s) of Fatigue	Population(s)	Statistical Findings (SMA)
**FSS** (M) [43]	MS and SLE	Werlauff et al., 2014 [39] Montes et al., 2015 [44] Giovannetti et al., 2016 [45] Dunaway Young et al., 2019 [13] Kizina et al., 2020 [17] Binz et al., 2021 [16]	**GF** **PF** **MoF**	Adults	Test–retest in SMA II, r = 0.98 (Werlauff et al., 2014 [39])
**PedsQL™ *MFS*** (M) ***PedsQL.org*** ***(accessed on 1 February 2023)*** [46,47,48]	Pediatric Patients	Montes et al., 2015 [44] Dunaway Young et al., 2019 [13]	**GF** **CF** **S/R-F**	Pediatrics and Adults (5–49 years) Parent-proxy (2–18 years)	N/A
**MFI** (M) [49]	Oncology and Chronic Fatigue Syndrome	Binz et al., 2021 and 2022 [16,22]	**GF** **PF** **MoF** **MF**	Adults	*General and Physical* *Fatigue* combined scale scores, Cronbach α = 0.84 (Binz et al., 2022 [22]) *General Fatigue* convergent validity with VAS, ρ = 0.7 (Binz et al., 2022 [22])
**PROMIS (F-SF)** (M) ***NIHpromis.org*** ***(accessed on 1 February 2023)*** [50]	Non-Disease Specific	Belter et al., 2020 [40]	**GF** **PF** **MoF** **MF**	Pediatrics and Adults (8–18+ years) Parent-proxy for younger patients	N/A
**OMNI (RPE)** (U) [51,52]	Non-Disease Specific	Bartels et al., 2020 [53] Cheng et al., 2022 [54]	**PF**	Adolescents and Adults (13–57 years)	N/A
**Fatigue Visual Analog Scale (VAS-F)** (U) [55]	Non-Disease Specific	Werlauff et al., 2014 [39] Binz et al., 2022 [22] van der Heul et al., 2022 [56]	**PF**	Adults (18+ years)	Test–retest in SMA II, r = 0.99 (Werlauff et al., 2014 [39])
**SMA-HI** (M) [57,58,59]	SMA	Zizzi et al., 2021 [59] Sansone et al., 2021 [57] Mazzella et al., 2022 [58]	**GF** **PF**	Adolescents and Adults (12–79 years)	Fatigue subscale, Cronbach’s α = 0.92 (Zizzi et al., 2021 [59]) Total score ICC = 0.86, Fatigue subscale ICC = 0.91 (Zizzi et al., 2021 [59])
***PROfuture*** (U) [38]	SMA	Domine et al., 2022 [38]	**PF**	Adolescents and Adults (14+ years)	N/A
**SMA-TOOL** (U) [21]	SMA	Vazquez-Costa et al., 2022 [37] Vazquez-Costa et al., 2022 [37]	**PF**	Pediatric and Adults (8+ years) Parent-proxy (2–8 years)	Perceived fatigability subscale, Cronbach’s α = 0.92 (Vazquez-Costa et al., 2022 [37])

U, Unidimensional; M, Multidimensional; MS, Multiple Sclerosis; SLE, Systemic Lupus Erythematosus; GF, General Fatigue; PF, Physical Fatigue; MoF, Motivational Fatigue; CF, Cognitive Fatigue; S/R-F, Sleep/Rest Fatigue; MF, Mental Fatigue; N/A: no statistical findings related to SMA.

## Data Availability

No new data was generated as part of this review.

## Abbreviations

SMA	Spinal Muscular Atrophy
SMN	Survival Motor Neuron
PROMs	Patient-Reported Outcome Measures
HRQoL	Health-Related Quality of Life
FSS	Fatigue Severity Scale
MFI	Multidimensional Fatigue Inventory
SMA-HI	Spinal Muscular Atrophy-Health Index
PedsQL™MFS	Pediatric Quality of Life Inventory™ Multidimensional Fatigue Scale
PROMIS F-SF	Patient-Reported Outcomes Measurement Information System Fatigue-Short Form
GCI-I	Clinical Global Impression-Improvement
VAS-F	Fatigue Visual Analog Scale
RPE	Rate of Perceived Exertion
NMJ	Neuromuscular Junction
CSF	Cerebrospinal Fluid
NCS-RNS	Nerve Conduction Study—Repetitive Nerve Stimulation
ATP	Adenosine Triphosphate
6MWT	Six-Minute Walk Test
6MMT	Six-Minute Mastication Test
ESTs	Endurance Shuttle Tests
ESWT	Endurance Shuttle Walk Test
ESNHPT	Endurance Shuttle Nine Hole Peg Test
ESBBT	Endurance Shuttle Box and Block Test
PFS	Pittsburgh Fatigability Scale
